# Spectrum and trends of cancer among HIV patients in Southwestern Uganda

**DOI:** 10.1371/journal.pone.0317222

**Published:** 2025-01-27

**Authors:** Raymond Atwine, Mitala Yekosani, Abraham Birungi, Brian Ssenkumba, Barbra Tuhamize, Richard Ezinga, Keneth Male, Taseera Kabanda

**Affiliations:** 1 Department of Pathology, Mbarara University of Science and Technology, Uganda; 2 Department of Microbiology, Mbarara University of Science and Technology, Uganda; 3 Department of Biochemistry, Mbarara University of Science and Technology, Uganda; Kisumu County, KENYA

## Abstract

**Background:**

Antiretroviral therapy (ART) restores cellular immunity, significantly reducing AIDS-related mortality and morbidity thus improving the quality of life among People living with HIV (PLHIV). Studies done in several countries show a decline in AIDS defining cancers (ADCs) with the introduction of ART however the increased longevity has led to the increase of Non-AIDS defining cancers (NADCs). The study was aimed at studying the changing spectrum and trends of cancer among Human Immunodeficiency Virus (HIV) patients in southwestern Uganda.

**Methods:**

The study was a retrospective chart review of records of HIV-positive patients attending/receiving care from the Oncology clinic and ISS clinic of Mbarara Regional Referral Hospital (MRRH) who were, diagnosed with cancer for the past 10 years (January 2012–2021). Data were statistically analyzed using STATA version 17 (Stata Corp, Texas, US) at *P* <  0.05.

**Results:**

Males were more common at 64.5% while the median age was 37 years (IQR 29–47 years). ADCs were seen in 77.5% of the population while participants with NADCs were older (p < 0.001). The majority 73.3% (283/386) were in later stages (3 and 4). Having either ADCs or NADCs was different across HIV stages (p < 0.001). The median baseline CD4 count was 205 cells/μl (IQR: 90–400 cells/μl). The median duration on ART was 15 months (IQR 3–65 months). Participants with ADCs had been on ART for a shorter duration of time (p < 0.001). Only the outcome of patients with ADCs were available. The outcome varied with sex (p < 0.036), baseline CD4 (p < 0.048), and HIV stage (p < 0.002). Males were more likely to die (30/38 or 78.95%) and lost to follow-up (26/41 or 60.98%). Participants with baseline CD4 cell count > 200 cells/μl were more than twice likely to be active in care. The Commonest ADC was Kaposi Sarcoma (KS) while the commonest NADC was Squamous cell carcinoma, Not otherwise specified. Age above 50 years was associated with a significantly reduced risk of ADCs (OR: 0.11; 95% CI: 0.03–0.43; p value: 0.002). The risk of ADCs increased from stage 2 (OR: 0.46, p-value: 0.03; 95% CI: 0.23–0.91) to stage 3 (OR: 1.13; p-value: 0.66; 95% CI: 0.65–1.97) but this was not statistically significant. The risk of ADCs decreased with increasing ART duration (*P* value < 0.05).

**Conclusion:**

ADCs are still a major health challenge in Southwestern Uganda despite the increasing the coverage and uptake of ART in region. These have mostly affected the young people, people who have been on HAART for a shorter period and those with lower CD4 cell count at initiation of ART.

## Introduction

To date, 79.3 million people have so far been infected by the Human Immunodeficiency Virus (HIV) [1]. Presently, 37.7 million people are living with HIV (PLHIV) globally. 25.4 million PLHIV (67%) are in Sub-Saharan (SSA). In Uganda, 1.5 million people are living with HIV, 5.4% being adults (15–49 years). With improvement in the coverage of Antiretroviral therapy (ART), there has been a significant decrease in the number of new infections, prevalence, and death globally [2]. Uptake of HIV prevention and treatment has improved over years in Uganda with ART coverage currently at 85% among adults, consequently reducing new infections from 94,000 in 2010 to 53,000 in 2019 which is still high [3,4]. HIV-related death has declined in Uganda from 53,000 in 2010 to 21,000 in 2019 with Non Communicable Diseases (NCDs) including cancers contributing a significant number to the mortality in addition to opportunistic infections [3,5,6].

HIV causes immunosuppression which increases the risk of developing cancer post infection with several viruses like Human Papilloma Viruses (HPV), Epstein Barr Virus (EBV), and Human Herpes Virus 8 (HHV-8), among others which are known to cause cancers [7]. Some of these cancers are collectively known as AIDS-defining cancers (ADCs) namely, Kaposi sarcoma (KS), Non-Hodgkin Lymphoma (NHL), and cancer of the cervix, and these can be the initial manifestation of AIDS [8–10]. However, these cancers are also seen in the general population though at a low prevalence [10–12]. HIV also increases the risk for other cancers collectively termed as non-AIDS defining cancers (NADCs), but not to the tune of ADCs [9]. A study conducted in Kyadondo County in central Uganda before the introduction of Antiretroviral Therapy (ART) revealed that KS, NHL, and cervical cancer were the commonest cancers (70%) seen among PLHIV. Studies have highlighted that low CD4 (<200 cells/mm³) count and high viral load (>500 copies/mL) increase the risk of almost all cancer [13,14].

The emergence of other cancers described as NADCs like conjunctival squamous cell carcinoma, breast carcinoma, hepatocellular carcinoma, and prostate cancer among others has been reported [15,16]. This rise of NADCs has been attributed to increased longevity of PLHIV, behavioral factors, environmental toxins, and the direct effect of HIV (activating several proto-oncogenes and inhibition of tumor suppressor gene) [17]. NADCs have reportedly superseded ADCs among AIDS patients in the United States [15,18] and Tanzania [19].

ART restores cellular immunity, significantly reducing AIDS-related mortality and morbidity thus improving the quality of life among PLHIV [20–22]. Studies done in several countries show a decline in ADCs with the introduction of ART [7,23,24]. The introduction of ART led to a decrease in the incidence of KS, and an increase in NHL and HPV-associated cancers including cervical cancer [25], however, some studies showed no effect on cervical cancer [16,26,27].

In Uganda, ART coverage has improved to 85% among adults of 15 years and over [4]. This study will assess the influence ART has had on trends and the spectrum of cancers among PLHIV in the last 10 years in Mbarara, southwestern Uganda. Mbarara is an area with the highest prevalence of HIV in Uganda and yet there is scarcity of data about cancer patterns among PLHIV in the region.

## Methods

### Study design and setting

The study was a retrospective chart review of records of HIV-positive patients attending/receiving care from the Oncology clinic and Immune Suppression Syndrome (ISS) clinic of Mbarara Regional Referral Hospital (MRRH) who were, diagnosed with cancer for the past 10 years (January 2012–2021). Records of patients under the age of 18 years and/or with missing data (ART regimen and baseline CD4 count) were excluded.

### Study procedures

**Fig 1:**
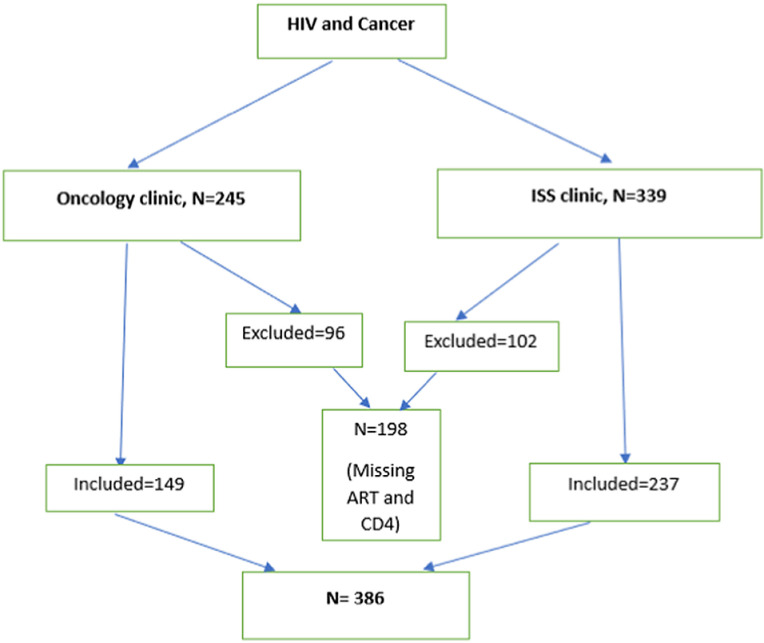
Study flow chart.

584 records of adult patients that attended ISS clinic oncology and were reviewed and sorted by the type of ART regimen and presence or absence of a cancer diagnosis.. The 584 patient files were assigned numbers from 1–584. These numbers were entered into STATA version 17. They were randomly redistributed and then picked as selected by the software. Those that did not meet the inclusion criteria were removed and replaced, until the 386 cases were achieved as seen in Fig 1. Cancer diagnosis was cross-checked with the records at the Pathology Laboratory and cancer registry to ensure quality and consistency of the data. Data on patient characteristics (age and gender), Baseline CD4 cell count (<200 and above 200), ART regimen at start (first or second line), duration on ART (≤6 months, 7–12 months, 13–24 months and > 24 months), Type and histological subtypes of cancer and HIV stage at cancer diagnosis were obtained. Cancer types were classified as either ADCs (KS, Cervical cancer, and NHL) or NADCs, and the clinical outcome was recorded as active, dead, loss to follow-up and transfer. The study was approved by Mbarara University Institutional Review Board (MUST-IRB) with study number (MUST-2022-681). Permission to access the data from Oncology and ISS clinics was also given by the respective unit heads.

### Data management and analysis

Data was entered in Epi Info software version 7 (CDC), transferred into Excel for cleaning, coded and analyzed using STATA version 17 (Stata Corp, Texas, US). Data was entered in Mbarara and stored in an encrypted database. The continuous variables were reported as medians, and discontinuous variables were reported as proportions and/or percentages. The dependent variables were the type and histological subtypes of cancer, and the independent variables included patient characteristics (age, gender), baseline CD4 cell count, baseline ART regimen, duration on ART till cancer diagnosis and HIV stage at cancer diagnosis. Chi-square tests was used to establish differences amongst established categories (ADCs and NADCs) and study participant characteristics. Regression analysis was used to establish the relationship between the ART regimen, duration on ART, HIV stage and the different cancer types in univariate and multivariate analyses. Results were reported as odds ratios and confidence intervals at 95%. A *p* < 0.05 was considered significant.

## Results

We reviewed 584 charts from the oncology and immunosuppression syndrome (ISS) clinics to determine participants with both cancer and HIV diagnosis as shown above. Charts that had both conditions were categorized based on whether the cancer type is classified as ADC or NADC. These were further scrutinized for mention of ART regimen and Baseline CD4 count. Those that were missing both the ART regimen and baseline CD4 were excluded from the study. Those that were included were 386.

### Baseline characteristics of study participants

Baseline characteristics of the patients’ reviewed records are summarized in Table 1. Overall, males were more than the females at 64.5% (249/386), the median age was 37 years (IQR 29–47 years), baseline CD4 count was 205 cells/μl, median duration of HAART was 15 months, more late stage HIV was documented and ADCs were more than NADCs at 77.5%. ISS clinic had only ADCs while Oncology had both ADCs and NADCs. Participants with NADCs were older (p < 0.001) and had been on HAART for a longer duration of time (p < 0.001). HIV stages were significantly different between the ADCs and NADCs.

**Table 1:** Baseline characteristics of study participants.

Characteristic	Total cohort (n %)	NADCS (n = 87)	ADCS (n = 299)	p-value
Sex (n %)				
Female	137 (35.5%)	38 (27.7%)	99 (72.3%)	0.070
Male	249 (64.5%)	49 (19.7%)	200 (80.3%)	
Age in years, median (IQR)	37 (29–47)	50 (42–57)	34 (28–41)	<0.001^*^
HIV stage (n %)				
Stage 1	52 (13.5%)	0 (0.0%)	52 (100.0%)	<0.001^*^
Stage 2	51(13.2%)	21 (41.2%)	30 (58.8%)	
Stage 3	144 (37.3%)	32 (22.2%)	112 (77.8%)	
Stage 4	139 (36.0%)	34 (24.5%)	105 (75.5%)	
Baseline CD4 Median(IQR).	205 (90–400)	278 (94–435)	200.5 (89–391)	0.085
ART Period in months, median (IQR)	15 (3–65)	47.5 (20–120)	8 (2–48)	<0.001^*^
Baseline ART Regimen, n (%)				
First Line	362 (97.3%)	82 (22.7%)	280 (77.3%)	0.843
Second line	10 (2.7%)	2 (20.0%)	8 (80.0%)	
Outcome			n (%)	
Active	51	–	51 (35.2%)	
Dead	38	–	38 (26.2%)	
Transfer	15	–	15 (10.3%)	
Lost to follow-up (LTF)	41	–	41 (28.3%)	

### Outcome compared with the different variables

Outcome (either active in care, dead, LTF, or transfer) are summarized in Table 2. The available records were for the 146 participants who had ADCs, and all these records were from the ISS clinic. The outcome of patients with NADCs were not available. The outcome varied with sex (p < 0.036), baseline CD4 (p < 0.048), and HIV stage (p < 0.002). More males were dead (30/38 or 78.95%) and lost to follow-up (26/41 or 60.98%) than females. Participants with baseline CD4 cell count >200 cells/μl were more active in care and with fewer deaths than their counterparts of less than 200 cells/μl. Advanced stages of HIV (stage 3 and stage 4) had majority of the participants across all outcomes.

**Table 2:** Outcome comparisons of study participant characteristics.

Variable	Outcome				P-value
	Active in care n (%)	Dead n (%)	LTFU n (%)	Transfer n (%)	
Sex					
Male	26 (50.98)	30 (78.95)	25 (60.98)	7 (46.67)	0.036^*^
Female	25 (49.02)	8 (21.05)	16 (39.02)	8 (53.33)	
Age category (years)					
18–24	2 (3.92)	5 (13.16)	4 (9.76)	2 (13.33)	0.315
25–49	38 (74.51)	30 (78.95)	33 (80.49)	12 (80.00)	
≥50	11 (21.57)	3 (7.89)	4 (9.76)	1 (0.67)	
Baseline CD4 (cells/μl)					
<200	12 (31.58)	14 (63.64)	14 (50.0)	6 (75.0)	0.048^*^
>200	26 (68.42)	8 (36.36)	14 (50.0)	2 (25.0)	
HIV stage					
Stage 1	6 (22.22)	6 (15.79)	10 (24.39)	4 (26.67)	0.002^*^
Stage 2	2 (7.41)	1 (2.63)	2 (4.88)	0 (0.00)	
Stage 3	10 (37.04)	17 (44.74)	11 (26.83)	6 (40.00)	
Stage 4	9 (33.33)	14 (36.84)	18 (43.90)	5 (33.33)	

### Spectrum/histological subtypes and of cancers among PLHIV in Southwestern Uganda

**Fig 2:**
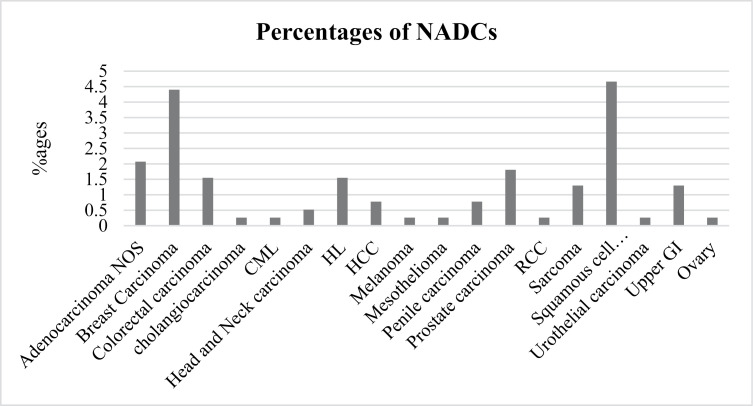
Bar graph showing proportion of NADCs.

ADCs are much more prevalent 77.5% (299/386) with KS the most observed ADCs (90%), followed by cervical cancer (8%), and NHL (2%). Among the NADCs, squamous cell carcinomas (sites not specified) were the majority (4.66%) (Fig 2).

### Trends of ADCs and NADCs over the 10-year period; and Trends of ADCs and NADCs with CD4 count

**Fig 3:**
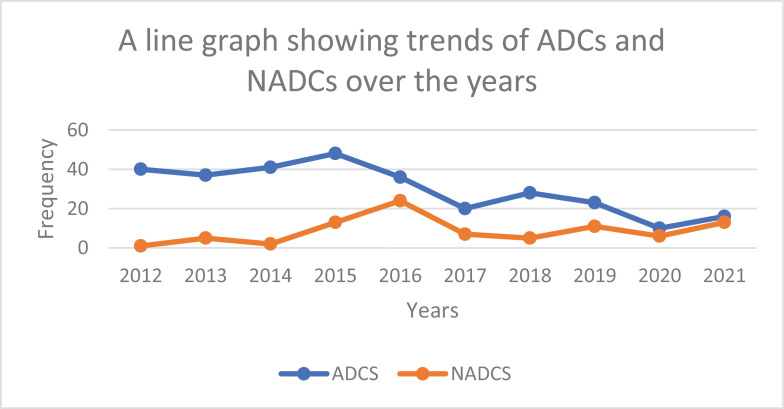
Line graph showing the trends of ADCs and NADCS over the 10 years period.

Generally, there is a steady decrease of ADCs and a steady fluctuation of NADCs over the 10-year period as seen in Fig 3.

**Fig 4:**
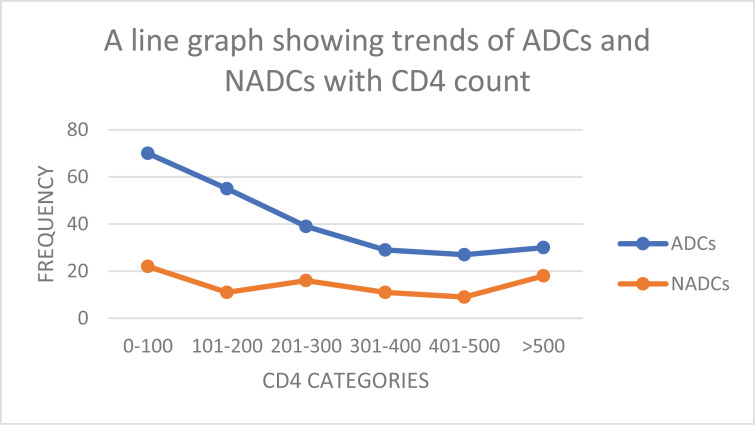
Line graph showing the trends of ADCs and NADCs with CD4 counts.

ADCs decrease with increasing CD4 count while NADCs remained relatively stable with the changing CD4 levels as seen in Fig 4.

### Influence of different drug regimens (combinations) and duration of HAART on cancers among those with ADCs

In both univariate and multivariate analysis, there was no significant relation between gender and ADCs. Age above 50 years was associated with a significantly reduced risk of ADCs in both univariate (OR: 0.07; 95% CI: 0.02–0.25; p value: <0.001) and multivariate analysis (OR: 0.11; 95% CI: 0.03–0.43; p value: 0.002). Because there were no participants with NADCs in stage 1 of HIV, we did not compare with the ADCs. At univariate analysis, the risk of ADCs increased from stage 2 (OR: 0.46, p-value: 0.03; 95% CI: 0.23–0.91) to stage 3 (OR: 1.13; p-value: 0.66; 95% CI: 0.65–1.97). Stage 4 was omitted in the analysis because of collinearity. In univariate analysis, baseline CD4 above 200 cell/μl was marginally associated with a reduced risk of ADCs with a p-value of 0.05, however, this was not significant at multivariate analysis (p-value 0.52). The risk of ADCs decreased with increasing ART duration in both univariate and multivariate analysis as seen in Table 3. There was no preferential distribution of ADCs in relation to ART regimen with p-values of 0.84 and 0.96 in univariate and multivariate analysis respectively.

**Table 3:** Effect of the different variables on ADCs in univariate and multivariate analysis.

Characteristic	Univariate analysis			Multivariate analysis		
	OR	P-value	95%CI	AOR	p-value	95%CI
Sex						
Male	1.57	0.071	0.96–2.55	1.16	0.64	0.63–2.15
Age categories						
25–49	0.60	0.415	0.17–2.05	0.67	0.55	0.17–2.25
50+	0.07	<0.001*	0.020–0.25	0.11	0.002*	0.03–0.43
HIV stage						
Stage1	1	0.03*	0.23–0.91			
Stage2	0.46	0.66	0.65–1.97			
Stage3	1.13					
Stage4	1					
Baseline CD4 categories						
>200	0.61	0.05^#^	0.37–1.01	0.82	0.52	0.45–1.50
ART Period in Months Categories						
7–12 Months	0.05	<0.0001*	0.01–0.20	0.06	<0.0001*	0.02–0.26
13–24 Months	0.06	<0.000*	0.02–0.23	0.06	<0.0001*	0.02–0.31
25+ Months	0.04	<0.0001*	0.01–0.12	0.05	<0.0001*	0.02–0.22
Baseline ARV line						
Second line	1.17	0.84	0.24–5.62	1.06	0.96	0.14–7.55

*P < 0.05.

^#^Marginally significant.

## Discussion

HIV-induced immune suppression increases the risk for both ADCs and NADCs. With over two decades of use of ART in Uganda, we expected that the number of PLHIV presenting with ADCs should be low. From this study, the number of PLHIV presenting or being managed for ADCs is still high (77.5%). KS is by far the most common ADCs (90%) seen to date followed by cervical cancer (8%) and NHL (2%) in that order. This is similar to recent studies done in Kampala that also reported KS as the most commonly reported cancer together with other ADCs [16,28]. The high KS prevalence is attributed to the high HIV prevalence in Mbarara district [29]. This high HIV prevalence of HIV is attributed to the fact that Mbarara is a trans-route city with lots of population but also the transactional sex practices, which allows even very poor people to have unsafe sex [30]. This is similar to sub-Saharan African countries who have also reported more ADCs as seen in Botswana, Kenya and Malawi [25,31,32]. However, this is different in developed countries where ADCs are much lower due to the low prevalence of HIV and improved health care behavior/systems in these countries [33]. Males make the biggest percentage of these patients. This is similar to studies done in the USA and China where males were significantly more than women [33–35] however most studies have contradictory findings [23,36]. We can attribute this to the poor health seeking behaviour of most men which causes these men to present to hospital with overtly symptomatic or advanced disease. Men are also more likely to die from ADCs which can be attributed to very late presentation for care. Men are also more likely to be less adherent to ART, they are also more likely to be lost to follow-up, and therefore less likely to suppress the viral load which increases their chances of getting ADCs [37–39]. Several NADCs were also noted among the reviewed charts including cancers of the breast, prostate, squamous cell carcinomas from other sites, HL among others. These can be attributed to other environmental risk factors and aging as seen in the general population [40–42].

We found that generally, there is a steady decrease of ADCs and a steady increase of NADCs over the 10-year period. This is similar to many post-ART studies that concur to this trend. The decrease of ADCs is due to improved efficacy and tolerability of ART [43]. ART access in resource limited settings has increased and ART also improved quality of life as demonstrated in Ugandan studies [44,45]. NADCs have increased in the post-ART era due to the increased longevity which increases the risk of environmental and age-related cancers (NADCs) [43,46].

Our study found that generally, ADCs decrease with increasing CD4 count while NADCs remained relatively stable with the changing CD4 levels. This is similar to many studies [47,48]. This is attributed to the fact that ADCs are influenced by the immunosuppression which is proportional to the CD4 count, while NADCs are not only affected by immunosuppression but by other factors (multifactorial), most of which include are infectious in etiology [49,50].

ADCs were also more likely to be found in the younger people of 49 years and below. Comparatively, NADCs were more in people above 50 years. This is similar to a study done in the US [43]. Age above 50 years was associated with a significantly reduced risk of ADCs compared to the younger people. The possible explanation to this is that older people are most likely to have been taking HAART for quite a long time, more adherent to ART and are more likely to have a suppressed viral load [38]. So the risk for ADCs is reduced but that of NADCs remains relatively similar to the general population due to factors like ageing [7]. Also the younger people are more likely to be involved in risky behaviours like alcohol intake, smoking among others [51]. This makes them likely to abandon care, skip taking their drugs, which causes non adherence hence increasing the risk for ADCs as it was observed in Zimbabwe [52].

Generally, the number of participants with either ADCs or NADCs rose with the increase in HIV stage. Stage 3 and 4 had significantly higher numbers of patients. This is expected because as one tends to AIDS, the risk of ADCs increases as described in a study done by Hernandez and Middleton [53]. In stage 1, there were no participants with NADCs. This hindered comparison with 52 participants who had ADCs in stage 1. Further, stage 4 of HIV was omitted in the analysis because of collinearity which is created by the natural progression of HIV. At univariate analysis, the median CD4 cell count for patients with ADCs was lower than that of participants with NADCs however there was no statistical significance when adjusted for other factors. A low CD4 cell count weakens the immune system, allowing neoplastic cells to proliferate unchecked and form masses in the body that are cancerous. This is comparable to several studies that have found a low CD4 cell count of less than 200 cells to be associated with a significantly increased cancer risk [9,23,27]. It is also similar to studies that showed higher CD4 count in NADCs [43].

Participants with ADCs had been on ART for a considerably shorter duration compared to the participants who had NADCs. This is similar to study done in US [43,47]. Duration of ART was the most important factor in determining the risk of ADCs. Even 6 months difference in the duration of ART creates a significant difference in the risk of ADCs. It is possible that participants with ADCs probably presented for treatment much later (with AIDS) due to symptoms related to cancer and not necessarily due to HIV infection-related symptoms. Therefore, shortly after the initiation of ART cancer was also diagnosed. Longer duration of ART leads to viral load suppression and the restoration of the immune system which protected these participants from ADCs as similarly seen in the US [47].

Furthermore, participants with longer ART durations are likely to live longer just like the rest of the general population and this would put them at the same risk of other cancers (NADCs) as also seen in several population studies [23]. As observed in this study, 97.3% (362/372) were on the first-line ART regimen and only 2.7% (10/372) were on the second-line regimen. These large differences in the number of participants couldn’t allow meaningful comparison of the categories.

### Study limitations and challenges

There was selection bias. This was minimized by using random computer-generated numbers.

## Conclusion and recommendations

ADCs are still a major health challenge in Southwestern Uganda despite the increasing coverage and uptake ART in region. These have mostly affected the young people living with HIV, people who have been on ART for a shorter period and those with lower CD4 cell count at initiation of ART.

We recommend that at the point of initiation of HAART, patients should be screened for mainly ADCs and adherence to HAART should emphasized.

Because of the limitations of the study design, we recommend a prospective study to further determine the prevalence of cancer among PLHIV and the interaction with the different ART regimens, CD4 cell count and viral load.

## Supporting information

S1 File Supporting information present shows the data collection form which was used during abstraction of data. (DOCX)
