# Supplementary material for: Spectrum and trends of cancer among HIV patients in Southwestern Uganda
Source: PLoS One. 2025 Jan 27;20(1):e0317222. doi: 10.1371/journal.pone.0317222 (PMC11771909; doi:10.1371/journal.pone.0317222)
Supplement: S1 File — (DOCX) [file pone.0317222.s001.docx]

**Supporting information**

**DATA COLLECTION FORM**

Participant Study ID: __________________________

**Participant Demographics:**

Gender/Sex: _________ Age: ________________ Hospital/Clinic Number: _________________

**Participant Clinical and HAART History:**

Place/Hospital/Clinic of HIV diagnosis: ___________________ HIV disease stage: _____________________

CD4 count at diagnosis (baseline CD4): ______________________

Baseline HAART Regimen: _______________________________

Date of Cancer diagnosis: ______________________

Date of HIV diagnosis: ______________________

Duration on HAART to cancer diagnosis: ______________________

Cancer diagnosis (type and histological subtype): ___________________________________________________________________________

**Clinical/Pathological Outcome (tick as applicable):**

Active Dead Loss to follow up Transferred
